# Analytical methods for determining environmental contaminants of concern in water and wastewater

**DOI:** 10.1016/j.mex.2024.102582

**Published:** 2024-01-24

**Authors:** Dana Kadadou, Lina Tizani, Habiba Alsafar, Shadi W. Hasan

**Affiliations:** a Center for Membranes and Advanced Water Technology (CMAT), Khalifa University of Science and Technology, PO Box 127788, Abu Dhabi, United Arab Emirates; b Department of Chemical and Petroleum Engineering, Khalifa University of Science and Technology, PO Box 127788, Abu Dhabi, United Arab Emirates; c Center for Biotechnology (BTC), Khalifa University of Science and Technology, PO Box 127788, Abu Dhabi, United Arab Emirates; d Department of Biomedical Engineering, Khalifa University of Science and Technology, PO Box 127788, Abu Dhabi, United Arab Emirates; e Emirates Bio-research Center, Ministry of Interior, Abu Dhabi, United Arab Emirates

**Keywords:** Analytical techniques, Water analysis, Emerging organic pollutants, Persistent organic pollutants, Inorganic compounds, Particulates, Microorganisms, Disinfection by-products, Review on analytical methods used for water and wastewater analysis.

## Abstract

Control and prevention of environmental pollution have emerged as paramount global concerns. Anthropogenic activities, such as industrial discharges, agricultural runoff, and improper waste disposal, introduce a wide range of contaminants into various ecosystems. These pollutants encompass organic and inorganic compounds, particulates, microorganisms, and disinfection by-products, posing severe threats to human health, ecosystems, and the environment. Effective monitoring methods are indispensable for assessing environmental quality, identifying pollution sources, and implementing remedial measures. This paper suggests that the development and utilization of highly advanced analytical tools are both essential for the analysis of contaminants in water samples, presenting a foundational hypothesis for the review. This paper comprehensively reviews the development and utilization of highly advanced analytical tools which is mandatory for the analysis of contaminants in water samples. Depending on the specific pollutants being studied, the choice of analytical methods widely varies. It also reveals insights into the diverse applications and effectiveness of these methods in assessing water quality and contaminant levels. By emphasizing the critical role of the reviewed monitoring methods, this review seeks to deepen the understanding of pollution challenges and inspire innovative monitoring solutions that contribute to a cleaner and more sustainable global environment.

• Urgent global concerns: control and prevention of pollution from diverse sources.
• Varied contaminants, diverse methods: comprehensive review of analytical tools.
• Inspiring a sustainable future: innovative monitoring for a cleaner environment.

## Graphical abstract

**Figure:**
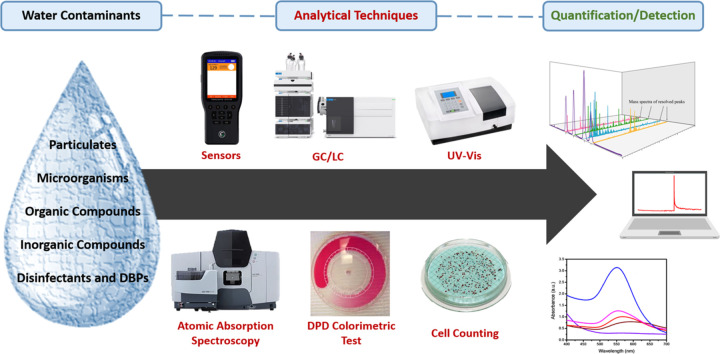

Specifications table

**Table:** 

Subject area:	Environmental Science
More specific subject area:	Water contamination
Name of the reviewed methodology:	Review on analytical methods for monitoring of water contaminants
Keywords:	Analytical techniques; water analysis; emerging organic pollutants; persistent organic pollutants; inorganic compounds; particulates; microorganisms; disinfection by-products.
Resource availability:	Not applicable
Review question:	Not applicable

**Method details**

## Introduction

Water pollution has become a critical global challenge, threatening the availability of safe and clean water resources for human consumption, agriculture, industry, and ecosystem preservation. Anthropogenic activities, such as industrial discharges, agricultural runoff, and improper waste disposal, introduce a wide range of contaminants into water bodies [1]. These pollutants include organic and inorganic compounds, particulates, microorganisms, and disinfectants and disinfection by-products (DBPs).In a study including 27 countries, river water and lake water were the most contaminated specifically with phtalate esters (PAEs), with Nigera shown as having the highest concentration of contaminants in water ressources [2], The presence of these pollutants in water bodies can impose severe health and environmental consequences, necessitating the implementation of effective monitoring methods to assess water quality, identify pollution sources, and take appropriate control measures. At the same time, water scarcity has emerged as a pressing concern worldwide, posing significant challenges to water availability and access [3]. As global population continues to grow, urbanize, and industrialize, the demand for water is also expected to increase substantially, while freshwater supplies remain limited and susceptible to depletion. Climate change also intensifies water scarcity, causing different precipitation patterns and more frequent and severe droughts. In regions experiencing water scarcity, the optimization of available water resources becomes vital, emphasizing the need for sensitive and efficient monitoring methods to ensure their sustainable and reasonable utilization.

In light of these interconnected challenges, the importance of monitoring methods for water quality assessment cannot be overstated. Robust and reliable analytical determination tools are necessary to detect and quantify pollutants accurately, identify pollution sources, and develop effective strategies to protect water resources [1]. By addressing water pollution through appropriate monitoring and analysis, the world will be working towards minimizing human contamination, promoting sustainable water management practices, and ensuring access to clean and sufficient water for present and future generations. Maintaining water quality is vital for preserving human health and the ecological balance of aquatic environments. The presence of pollutants in water sources can lead to severe health consequences when consumed or come into contact with, making it a top priority to detect and quantify these contaminants accurately and in a timely manner. Additionally, water scarcity exacerbates the challenge, as it necessitates the optimization of available water resources, emphasizing the need for precise and efficient monitoring methods to ensure their safe utilization.

Consequently, several analytical methods have been developed for the determination of pollutants in water. One of the most powerful analytical tools utilized in analytical chemistry is chromatography, which allows for qualitative and quantitative analysis, as well as separation under defined conditions. Chromatography can generally be categorized into gas chromatography (GC) and liquid chromatography (LC), depending on the nature of the mobile phase used. Given that GC suffers from several drawbacks related to time consuming procedures, LC has replaced GC in several applications. Thus, there has been rapid development in LC technologies, leading to enhanced instruments like high-performance liquid chromatography (HPLC), which is widely used today and offers several advantages including improved sensitivity, specificity, cost-effectiveness.

This paper aims to explore and critically evaluate the diverse array of analytical determination tools used for monitoring and analyzing water samples contaminated with organic compounds, inorganic compounds, particulates, microorganisms, disinfectants, and disinfection by-products. By highlighting the importance of monitoring methods, the focus is to seek to underscore their role in assessing water quality, understanding contamination patterns, and devising appropriate strategies for mitigating pollution and preserving this invaluable resource for future generations. Through a comprehensive review of these analytical techniques, the goal is to aspire to foster a deeper understanding of water quality challenges and inspire the advancement of innovative monitoring solutions that can help secure clean and sustainable water supplies worldwide.

## Analytical techniques

Different analytical techniques have been developed and applied for the detection of various groups of water contaminants. In the detection of these contaminants in water, sample preparation plays a crucial role, often involving clean-up and pre-concentration steps mandatory for the subsequent analytical procedures. Two techniques are used for sample preparation, liquid-liquid extraction (LLE) and solid phase extraction (SPE). LLE uses two immiscibles liquids, typically one aqueous and one organic, in order to separate compounds. LLE is valued for its versatility in handling a wide range of analytes and sample matrices. On the other hand, the principle of SPE involves the extraction of analytes from a complex matrix, and is based on transferring the analyte to the solid phase in the first step and then isolating it from the sample. This is followed by the recovery of the analytes back into the initial phase using a suitable solvent to allow for analysis. LLE typically involves lower equipment costs compared to SPE, but one significant drawback of LLE is its relatively high consumption of organic solvents. This makes SPE the most commonly used pioneering technique that consists of the extraction of analytes from a complex matrix and is designed for rapid, selective sample preparation and purification prior to the chromatographic analysis (e.g. HPLC, GC, TLC) [4]. Commercially available cartridges are commonly used in this technique. The choice of the sorbent is of high importance to achieve a high recovery rate. However, SPE presents some disadvantages correlated to channeling phenomena as well as blockage of the cartridges especially in complex media. This has paved the way for researchers to explore new ways to enhance this technique.

One improvement in SPE involves using discs instead of cartridges. The discs function in a similar way as cartridges, allowing the sample to pass through the device. For large volume samples, such as those encountered in environmental samples, the discs are advantageous over cartridges. Discs provide shorter processing times due to their large cross-sectional area and ease interaction with analytes. Moreover, their smaller particles size and high mechanical stability reduce channeling, a common drawback of cartridges [5]. Another area of research focus is the development of sorbents for SPE. Due to their unique physicochemical and mechanical properties, carbon nanostructures are a material of choice to be used in SPE operations. Carbon nanotubes (CNTs) are extensively researched and can be used as discs, or combined with different nanoparticles, such as magnetic particles, and can be integrated into microextraction devices. Tomai et al. developed a novel SPE technique based on nanoporous oxidized CNT membranes. These membranes can be designed in different sizes and shapes and consist entirely of sorbent materials. This device is particularly suitable for environmental samples. In pesticide analysis, in addition to the sample preparation and analysis methods mentioned for organic compound, new techniques have emerged. For this category, sample preparation is often the most critical and time-consuming step. Various extraction methods are employed, starting from conventional techniques like solid-phase extraction (SPE) and liquid-liquid extraction (LLE, commonly utilized for concentrating analytes and eliminating matrix interference [6]), to more sophisticated approaches that demand reduced solvent usage. These advanced techniques include dispersive solid-phase extraction (dSPE), stir bar sorptive extraction (SBSE), magnetic solid-phase extraction (MSPE), solid-phase microextraction (SPME), and dispersive liquid-liquid microextraction (DLLME). The principle of dispersive solid-phase extraction (dSPE) is based on adding a sorbent into the analytical solution, followed by dispersion. In this process, the sorbent is separated from the analytes by centrifugation or filtration. dSPE offers the advantages of simplicity and fast treatment time. Furthermore, the principle of magnetic solid-phase extraction (MSPE) is based on the target analyte being adsorbed on a magnetic adsorbent, which is then separated by an external magnetic field. This technique offers high extraction efficiency along with a high extraction capacity. Moreover, solid-phase microextraction (SPME) has the advantages of being a simple method for preparing samples with the added benefit of using a low solvent, while being sensitive since, in this method, the analyte is being distributed between the bulk and the fiber coating [7]. Another new technique, liquid-liquid microextraction (DLLME), has been developed, which uses microliters of extraction solvent. Nevertheless, DLLME also has some disadvantages, such as the non-environmentally friendly nature of the extraction solvents and the need for premixing before injection, limiting its applicability. Furthermore, deep eutectic solvents (DES) have emerged as alternatives to traditional solvents due to their low volatility, thermal stability, and low vapor pressure [8]. The solvent bar microextraction technique involves using a hollow fiber with one sealed end. This tube is immersed in the agitated samples. This configuration minimizes solvent loss, and the extracted solvent is subsequently analyzed using GC equipped with an electron detector. Another innovative technique for pesticide detection in water is chemiluminescence /flow injection (CL/FI). The photochemically induced photoluminescence method uses light as a reagent, and thus eliminates the need for polluting reagents that can harm the environment. Additionally, it offers the advantages of enhanced selectivity, sensitivity, and reaction time. Furthermore, the flow injection setup consists of two points: injection and detection, with the analyte undergoing a physical or chemical transformation in between. This is achieved by injecting a specific volume of the sample using a carrier solution to a flow-through detector. This technique is both simple and inexpensive due to the fact that it employs basic pumps and low-pressure valves. Coupling FI with CL offers the advantage of using the same instrumentation while allowing for the monitoring of light emission from CL reactions with controlled irradiation times.

In organic compounds, chromatography is one of the most commonly used analytical methods for separating a mixture of components based on the differences in their rates of passage through a liquid or gaseous phase. Chromatography provides both qualitative and quantitative results. In GC, a chemically inert gas serves as the mobile phase, while in LC, a liquid solvent is used. One of the main advantages of GC is that it requires only a small amount of the sample in order to separate complex mixtures. Nonetheless, GC suffers from several drawbacks including the use of durable solvents for extraction and the use of potentially explosive esterification reagents. Due to these limitations, GC has been replaced by LC in many cases. Conventional LC methods are characterized by stationary phases like C8 and C18, particle size ranging from 3 to 5 µm, as well as column lengths that vary between 10 and 25 cm. Moreover, new chromatographic techniques are emerging as advancements in miniaturized chromatographic methods. These techniques are based on reducing particle size or the diameter of the inner LC column, enabling them to achieve better performance in terms of sensitivity, efficiency, peak resolution, and shorter analysis times compared to conventional methods.

The advancement of LC methods has led to HPLC, which employs chromatographic columns with small packing particle sizes, typically in the range of 3–10 µm, in order to enhance the efficiency of chromatographic separations. This requires high pressure to allow and maintain suitable flowrates within the system. Nowadays, HPLC is one of the most versatile and commonly used chromatographic methods. Ultra-pure liquid chromatography (UPLC) has demonstrated higher resolution, along with both increased and decreased response times, achieved through the use of particle sizes of 1.7 µm under high pressure operating conditions [9]. Another technology, capillary liquid chromatography (CLC), is based on employing columns of 500 µm internal diameter, leading to lower sample requirements and reduced flowrates. Consequently, this approach minimizes solvent consumption and enhances chromatographic sensitivity. CLC enables efficient analysis with flowrates in the range of µL/min. Recent studies utilizing various chromatography techniques to determine fluorinated compounds in river waters [10] have shown that UHPLC and PLC outperform HPLC in terms of analysis time and resolution. UHPLC-MS/MS provides better MLDs compared to the other techniques. Techniques employing MS/MS detectors (UHPLC-MS/MS and HPLC-MS/MS) have shown better selectivity than those using MS detectors. Nonetheless, this is associated with higher costs. One widely used analytical methods for determining analytes in water is based on liquid chromatography coupled with mass spectrometry (LC-MS) or tandem mass spectrometry (LC-MS/MS). Generally, SPE is employed prior to these approaches. While these methods are well-established, there are still several disadvantages that need to be addressed, such as resolution issues [10]. These analytical methods are generally used for organic compound detection that covers persistent organic polluants (POPs), including pesticides, as well as emerging organic polluants (EOPs). A novel approach has been recently developed for antibiotic decomposition such as Trimethoprim, this method is based on Photocatalytic activation of persulfate (PS) and has been proven to be effective and environmently friendly. It used CuFe-layered double hydroxide (LDH) coated graphene oxide (CuFe-LDH/ GO) composite [11].

While GC, coupled to mass spectrometry (GC-MS,GC-MS/MS,HRMS, etc,), was the most conventional method for analyzing pesticides in water in the 1990s, advancements in the GC-MS method, based on atmospheric pressure ionization sources, have enhanced the capability for analyzing pesticide residues in environmental matrices while still achieving the required detection limits for analytes (in the µg/L range) [12]. Moreover, for pesticides with more polar structures and low volatility, analyzing with GC-MS is not efficient without a prior derivatization process. In these cases, Liquid Chromatography Mass Spectrometry (LC-MS) has been employed for the analysis [13].

In inorganic compound analysis, atomic absorption spectrometry is mainly used for the analysis of metallic elements, employing a radiant beam with a specific wavelength passing through the material. This analytical technique determines the concentration of a specific element in a sample by leveraging the principle that atoms (and ions) absorb light at a unique wavelength [14]. In contrast, atomic emission spectrometry relies on the emission of characteristic wavelengths. This method involves heating atoms in the gas phase, causing electrons to transition to higher energy levels and subsequently emit wavelengths specific to each element [14]. Calorimetry serves as a valuable quantitative analysis tool, known for its high sensitivity, good selectivity, accuracy, and wide range of applications. The underlying working principle involves measuring the absorption of radiant energy by a strongly colored solution at a specific wavelength, enabling the determination of the concentration of a specific compound [14].

UV-Vis spectroscopy serves as a qualitative and quantitative method used for the determination of certain inorganic compounds capable of absorbing wavelengths in the ultraviolet and visible light spectrum. Fluorimetry, based on measuring fluorescence at a specific wavelength, provides quantitative results, while chemiluminescence relies on the emission of a certain wavelength when the excited species returns to the ground state, using energy acquired from chemical reactions. Furthermore, ionic chromatography (IC) is commonly used for detecting ions in aqueous samples. This technique offers the advantages of lower detection limits, small sample aliquots, and the elimination of interferences that other techniques suffer from. The working principle involves injecting a sample into a liquid mobile phase, which is then pumped through two ion exchange columns, namely, separator and suppressor. These two columns are inserted in series in the system [14].

In contrast, particulate pollutants typically lend themselves to simpler analytical techniques for qualitative and quantitative detection. On the other hand, microorganisms present a distinct category of pollutants, demanding precise and prompt monitoring in water bodies. This is of high importance for establishing a robust surveillance system, particularly for microorganisms that may present health risks [3]. Conventional analytical techniques primarily revolve around the identification of specific microorganism components. These conventional approaches can be broadly classified into molecular biology methods, culture-based methods, and immunology-based methods. Over the years, a multitude of methods falling within these categories have been employed for microorganism detection.

Moving on to disinfectants and disinfection by-products (DBPs), which utilize an array of chromatographs, such as GC, IC, and HPLC, to determine their concentrations in water. Among these, direct headspace methods, which work by extracting and concentrating volatiles from debris, has emerged as a prominent choice due to its reliability, even though it requires higher sample dosage and may prioritize consistency over absolute accuracy. Besides analyzing samples through precursors, new methods known as micro-solid phase extraction techniques have been established to facilitate analysis through solid samples. As such, multiple methods including GC-MS, Gas Chromatography combined with Electron Capture Detection (GC-ECD), Ion Chromatography Electrospray Ionization tandem Mass Spectrometry (IC-ESI-MS) and Ion Chromatography with Conductivity Detection (IC-CD) have been utilized for the diagnosis of DBPs in water. All in all, the merged application of separation techniques and ionization methods has indeed simplified the identification of numerous types of DBPs in water. While GC is considered a gold standard in DBPs identification, GC-MS faces challenges in the detection of polar and thermally unstable DBPs. In such cases, ESI is being coupled with LC.

## Organic compounds

**Fig. 1:**
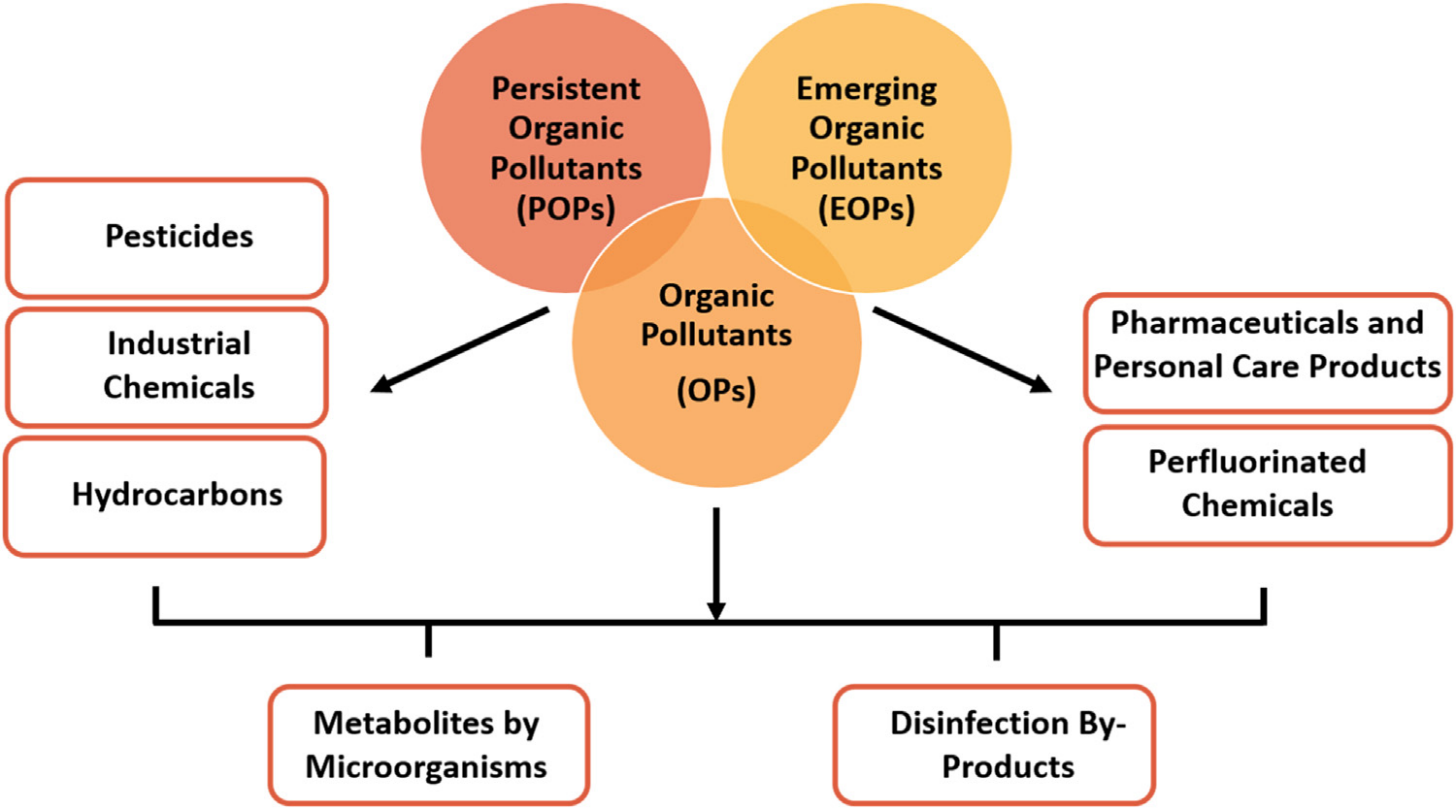
Major classes of organic pollutants, focusing on persistent organic pollutants (POPs) and emerging organic pollutants (EOPs).

Organic pollutants in water are chemical compounds of organic origin, natural or synthetic in nature, that can harm the environment and human health. There are several routes through which these compounds can occur in water bodies. Naturally occurring organic compounds are often produced by living aquatic microorganisms in lakes, rivers and oceans for purposes that include communication, defense, and energy storage. Examples of these naturally occurring organic compounds are 2-methylisoborneol and geosmin. Such occurrences are of concern due to their apparent toxicity in aquatic environments. Other organic compounds are synthetic in nature, and commonly source from industrial activities and flow into and contaminate water bodies. As a result, volatile organic compounds (VOCs), pesticides, phenolic compounds, phthalates, and nitrogen-containing compounds have been identified as water pollutants. VOCs can easily evaporate into the air and can have a negative impact on the environment and human health. Unfortunately, VOCs are also identified as persistent organic pollutants (POPs) in the aquatic environment. Similarly, the combustion of organic compounds produces dioxins and polynuclear aromatic hydrocarbons (PAHs). These compounds can be detected in surface waters due to their discharge from industrial waste. In addition, the disinfection of treated water can result in the presence of chlorination by-products in drinking water. Therefore, there are strict regulations by the World Health Organization (WHO) to limit the presence of organic constituents due to their negative impact on the environment and human health. The analytical techniques used for the identification and quantification of organic compounds. The subsequent sections will list organic pollutants which are grouped under persistent organic pollutants (POPs) and emerging organic pollutants (EOCs), as shown in Fig. 1.

### Persistent organic pollutants (POPs)

POPs are a group of chemical compounds that are highly persistent in the environment. In other words, they do not break down easily and can persist in the environment for many years. As a result, they pose a threat on human health and the environment. POPs include a wide range of chemicals including aromatic hydrocarbons such as benzene, xylene, and ethylbenzene which are frequently released from petroleum refineries. These pollutants have mutagenic, carcinogenic, immunotoxic, and teratogenic effects on both lower and higher forms of life. Many pesticides, especially organochlorine pesticides (OCPS) are classified as POPs as well. Several OCPs have been used in the agriculture to kill unwanted pests and promote crop yield in recent decades. Consequently, these pesticides have been easily introduced as contaminants into the environment. Additionally, it has been noted that several metabolites of DDT such as chlordane, toxaphene, and biphenyl products have been generated unintentionally, adding up to the produced toxic contamination. Moreover, dichloroethylene, trichloroethylene, and dioxin among others are considered POPs which are products of industrial activities. Such POPs have been heavily utilized during the industrial revolution period while others have been inadvertently produced through the incineration of medical waste, industrial processes that release smoke, or other means. In general, POPs can be classified into three types, namely hydrocarbons, pesticides, and industrial chemicals. Table 1 shows the most common controlled classes of POPs along with their guideline limits, and detection techniques.

**Table 1:** Common persistent organic pollutants classes, names, allowable drinking water and effluent levels, and analytical detection techniques.

Class	Name	Drinking water EPA maximum contaminant level goal (mg/L) [1]	Surface water EPA maximum recommended level (µg/L) [3]	Analytical technique	Ref.
Hydrocarbons	Benzene	0	16–58	Solid phase microextraction technique followed by GC-MS	[4]
	Toluene	1	520	Solid phase microextraction technique followed by GC-MS	[4]
	Xylenes	10		Solid phase microextraction technique followed by GC-MS	[4]
	Polycyclic aromatic hydrocarbons	0		Solid phase microextraction technique followed by GC-MS	[4]
	Alachlor	0.002		GC-MS	[5]
	Atrazine	0.003		Oxidized buckypaper (BP) as a sorbent membrane of a stir-disc solid phase extraction module	[9,10]
	Ethylbenzene	0.7		Solid phase microextraction technique followed by Gas Chromatography mass spectrometry (GS-MS)	
Pesticides	Carbofuran	0.04		1- LLE and a TPI on-column GC/MS method 2- LC 3- LC–MS with electrospray ionization (ESI) 4- Reversed phase liquid chromatography (RP-HPLC)	[6,[14], [15], [16]
	2,4-D	0.07	12,000	1- HPLC 2- Reversed phase liquid chromatography (RP-HPLC)	[16], [17], [18], [19], [20]
	Dalapon	0.2		1- GC 2- IC-MS	[21], [22], [23]
	Dinoseb	0.007		1- HPLC 2- GC 3- Capillary electrophoresis 4- Electrochemical methods and kinetic-spectrophotometry	[16,[24], [25], [26], [27], [28], [29]
	Diquat	0.02		1- Reversed phased liquid chromatography (HPLC) with UV detection 2- Liquid chromatography - (electrospray ionization) mass spectrometry [LC-(ESI)MS] 3- LC-MS 4- HPLC-MS/MS	[30], [31], [32], [33]
	Endothall	0.1		1- GC-MS 2- IC-MS	[34], [35], [36]
	Endrin	0.002	0.03	GC	[37]
	Glyphosate	0.7		1- LC-MS/MS 2- HPLC with fluorescence detection 3- HPLC with UV detection 4- HPLC-MS/MS 5- GC-MS/MS 6- GC with detection by flame photometry (DFC)	[38,39]
	Lindane	0.0002	4.4	1- Solvent bar microextraction technique using GC 2- GC	[37,40]
	Methoxychlor	0.04	0.02	GC	[37]
	Oxamyl	0.2		1- Photo-induced chemiluminescence (PICL) detection with FI methodology 2- LC–MS with electrospray ionization (ESI) 3- CEC-atmospheric pressure photoionization (APPI)-MS system	[8,^14^,41]
	Picloram	0.5		Reversed phase liquid chromatography (RP-HPLC)	[16]
	Simazine	0.004		1- PES microextraction followed by LC-MS/MS 2- LC–MS with electrospray ionization (ESI) 3- Reversed phase liquid chromatography(RP-HPLC)	[9,^14^,16]
	Toxaphene	0.003	7E-4	GC	[37]
	2,4,5-TP (Silvex)	0.05	400	Reversed phase liquid chromatography (RP-HPLC)	[16]
	Chlordane	0.002	3.2E-4	GC	[37]
	Hexachlorobenzene	0.001	7.9E-5	GC	[37]
Industrial Chemicals	Polychlorinated biphenyls	0.0005	6.4E-5	1- Solid phase microextraction (SPE) and GC 2- Solid phase extraction followed by GC/MS 3- Gas chromatographic electron capture mass spectroscopy (GC-ECMS) 4- SPE and GC-MS 5- High resolution gas chromatography/high resolution mass spectrometry (HRGC/HRMS)	[42], [43], [44], [45], [46]
	Carbon tetrachloride	0.005	5		
	o-Dichlorobenzene	0.6	3000		
	p-Dichlorobenzene	0.075	900	Capillary GC-MS	[47]
	1,2-Dichloroethane	0	650		
	1,1-Dichloroethylene	0.007	20,000		
	cis-1,2-Dichloroethylene	0.07			
	trans-1,2-Dichloroethylene	0.1	4000		
	1,2-Dichloropropane	0	31		
	Epichlorohydrin	0			
	1,1,2-Trichloroethane	0	8.9	Capillary GC-MS	[47]
	Dioxin (2,3,7,8-TCDD)	0.00000003	5.1E-9		

### Emerging organic pollutants (EOPs)

Emerging organic pollutants (EOPs) are a diverse group of synthetic and naturally existing organic pollutants that have been recently detected in water, but are still not regulated under existing environmental legislation. Because EOPs are generally challenging to track, they are not included in water monitoring programs. It is, therefore, anticipated that EOPs will eventually build up in water bodies if left unregulated [3]. The primary threats linked to EOPs and their by-products are due to the lack of extensive research on the environmental and human toxicity of the majority of these compounds. Research has shown, however, that EOPs could pose threats on human health.

The major classes of EOPs include pharmaceuticals, pesticides, perfluorinated substances, and microplastics. In this review, pesticides and microplastics have been discussed in Sections "Persistent organic pollutants (POPs)" and "Microorganisms". Thus, this segment will focus on pharmaceuticals such as antibiotics and anti-inflammatories, and perfluorinated substances. Table 2, Table 3, Table 4 list those compounds along with their chemical structures and analytical methods.

**Table 2:** Common antibiotics classes, chemical structures, and analytical detection techniques.

Class	Name	Structure	Analytical technique	Refs.
Macrolides	Roxithromycin		1- LC with coulometry 2- LC with electrochemical detection 3- LC with UV detection	[48], [49], [50], [51]
	Erythromycin		1- LC-MS coupled with the calibration technique of isotope dilution 2- LC with electrochemical detection	[48,52]
Tetracyclines	Tetracycline		1- UHPLC with fluorescence and tandem mass spectrometry detection 2- Solid-phase extraction and LC-MS 3- Laponite-based dual-channel fluorescent nanoprobe 4- Raman fingerprint strip sensor 5- HPLC coupled with DAD (diode array detection) 6- Glutathione (GSH)-protected fluorescent Au nanoclusters (GSH-AuNCs) 7- LC-MS/MS	[53], [54], [55], [56], [57], [58], [59]
	Oxytetracycline		1- UHPLC with fluorescence and tandem mass spectrometry detection 2- Solid-phase extraction and liquid chromatography–tandem mass spectrometry 3- HPLC coupled with DAD (diode array detection) 4- Raman fingerprint strip sensor 5- LC-MS/MS	[53,^54^,^56^,^57^,59]
	Chlorotetracycline		1- UHPLC with fluorescence and tandem mass spectrometry detection 2- Solid-phase extraction and liquid chromatography–tandem mass spectrometry 3- HPLC coupled with DAD (diode array detection) 4- Raman fingerprint strip sensor 5- LC-MS/MS	[53,^54^,^56^,^57^,59]
	Doxycycline		1- UHPLC with fluorescence and tandem mass spectrometry detection 2- Solid-phase extraction and liquid chromatography–tandem mass spectrometry 3- HPLC coupled with DAD (diode array detection)	[48,^53^,^54^,56]
Fluoroquinolones	Ofloxacin		1- Solid-phase extraction (SPE), followed by liquid chromatography electrospray ionization tandem mass spectrometry (LC-MS/MS) 2- HPLC with photoinduced fluorimetric (PIF) detection 3- ultra performance liquid 4- Chromatography electrospray tandem mass spectrometry (UPLC-MS/MS) 5- HPLC with UV detection	[60], [61], [62], [63]
	Ciprofloxacin		1- LC-MS coupled with the calibration technique of isotope dilution. 2- HPLC-MS 3- PES followed by LC-MS/MS 4- HPLC with photoinduced fluorimetric (PIF) detection 5- Ultra performance liquid chromatography electrospray tandem mass spectrometry (UPLC-MS/MS) 6- HPLC with UV detection 7- Lyophilization combined with LC-MS/MS	[9,^52^,[60], [61], [62],^64^,65]
	Norfloxacin		1- PES followed by LC-MS/MS 2- HPLC with photo-induced fluorimetric (PIF) detection 3- Ultra performance liquid chromatography electrospray tandem mass spectrometry (UPLC-MS/MS) 4- HPLC with UV detection 5- Lyophilization combined with LC-MS/MS	[9,[60], [61], [62],65]
	Enoxacin		1- HPLC with photoinduced fluorimetric (PIF) detection 2- Ultra performance liquid chromatography electrospray tandem mass spectrometry (UPLC-MS/MS) 3- HPLC with UV detection 4- Lyophilization combined with LC-MS/MS	[60], [61], [62],65]
	Enrofloxacin		Ultra performance liquid chromatography electrospray tandem mass spectrometry (UPLC-MS/MS)	[61]
Sulfonamides	Sulfamethizole		1- Electrochemical sensor based on an electropolymerized molecularly imprinted polymer (MIP) film 2- HPLC- DAD	[66,67]
	Sulfathiazole		1- Lyophilization combined with LC-MS/MS HPLC- DAD 2- LC-MS/MS 3- UHPLC–MS–MS	[59,^65^,68]
	Sulfamerazine		1- Lyophilization combined with LC-MS/MS 2- HPLC- DAD 3- HPLC-FLD	[65,^67^,^69^,70]
	Sulfaquinoxaline		1- Oxidized buckypaper (BP) as a sorbent membrane of a stir-disc solid phase extraction module 2- Lyophilization combined with LC-MS/MS	[10,65]
	Sulfamonomethoxine		UHPLC–MS–MS	[68]
	Sulfapyridine		1- HPLC- DAD 2- UHPLC–MS–MS	[67,68]
Chloramphenicols	Sulfamethoxazole		1- Solid-phase extraction (SPE), followed by liquid chromatography electrospray ionization tandem mass spectrometry (LC-MS/MS) 2- LC-MS coupled with the calibration technique of isotope dilution 3- HPLC 4- PES followed by LC-MS/MS 5- Oxidized buckypaper (BP) as a sorbent membrane of a stir-disc solid phase extraction module 6- Ultra performance liquid chromatography electrospray tandem mass spectrometry (UPLC-MS/MS) 7- Lyophilization combined with LC-MS/MS 8- HPLC-FLD 9- LC-MS/MS	[9,^10^,^52^,^59^,^61^,^64^,^65^,[69], [70], [71]
	Sulfadiazine		1- PES followed by LC-MS/MS 2- Oxidized buckypaper (BP) as a sorbent membrane of a stir-disc solid phase extraction module 3- Lyophilization combined with LC-MS/MS 4- HPLC- DAD 5- HPLC-FLD 6- LC-MS/MS 7- UHPLC–MS–MS	[9,^10^,^59^,^65^,^67^,^68^,70]
	Trimethoprim		1- LC-MS coupled with the calibration technique of isotope dilution 2- PES followed by LC-MS/MS 3- Ultra performance liquid chromatography electrospray tandem mass spectrometry (UPLC-MS/MS)	[2,^9^,^11^,^52^,^61^,72]
	Chloramphenicol		1- UHPLC-MS/MS 2- Mini solid phase extractor (MSPE) with HPLC 3- LC-MS/MS	[73,74]
	Thiamphenicol		LC-MS/MS	[74,75]
	Florfenicol		LC-MS/MS	[74,75]

**Table 3:** Common NSAIDs, chemical structures, and analytical detection techniques.

NSAID	Structure	Analytical technique	Ref.
Diclofenac		1- Solid-phase extraction (SPE), followed by liquid chromatography electrospray ionization tandem mass spectrometry (LC-MS/MS) 2- LC-MS coupled with the calibration technique of isotope dilution 3- HPLC-MS 4- Magnetic solid phase extraction method (MSPE) using magnetic cellulose nanoparticles (MCNPs) 5- PES followed by LC-MS/MS 6- Oxidized buckypaper (BP) as a sorbent membrane of a stir-disc solid phase extraction module	[9,^10^,^52^,^64^,^71^,76]
Ibuprofen		1- Solid-phase extraction (SPE), followed by liquid chromatography electrospray ionization tandem mass spectrometry (LC-MS/MS) 2- LC-MS coupled with the calibration technique of isotope dilution 3- HPLC-MS 4- Magnetic solid phase extraction method (MSPE) using magnetic cellulose nanoparticles (MCNPs) 5- Oxidized buckypaper (BP) as a sorbent membrane of a stir-disc solid phase extraction module	[52,^64^,^71^,^76^,77]
Ketoprofen		1- Solid-phase extraction (SPE), followed by liquid chromatography electrospray ionization tandem mass spectrometry (LC-MS/MS) 2- HPLC-MS 3- PES followed by LC-MS/MS 4- Oxidized buckypaper (BP) as a sorbent membrane of a stir-disc solid phase extraction module	[9,^10^,^64^,71]
Naproxen		1- Solid-phase extraction (SPE), followed by liquid chromatography electrospray ionization tandem mass spectrometry (LC-MS/MS) 2- LC-MS coupled with the calibration technique of isotope dilution 3- HPLC-MS 4- Magnetic solid phase extraction method (MSPE) using magnetic cellulose nanoparticles (MCNPs) 5- Oxidized buckypaper (BP) as a sorbent membrane of a stir-disc solid phase extraction module	[10,^52^,^64^,^71^,76]
Acetylsalicylate Acid			
Salicylic Acid		1- Solid phase extraction followed by liquid chromatography-electrospray ionization-tandem mass spectrometry (LC-ESI-MS/MS) 2- Oxidized buckypaper (BP) as a sorbent membrane of a stir-disc solid phase extraction module	[10,78]
Acetaminophen		1- Solid-phase extraction (SPE), followed by liquid chromatography electrospray ionization tandem mass spectrometry (LC-MS/MS) 2- LC-MS coupled with the calibration technique of isotope dilution 3- Solid phase extraction followed by liquid chromatography-electrospray ionization-tandem mass spectrometry (LC-ESI-MS/MS) 4- PES followed by LC-MS/MS 5- Oxidized buckypaper (BP) as a sorbent membrane of a stir-disc solid phase extraction module	[9,^10^,^52^,^71^,78]
Indomethacin		1- HPLC with UV detection 2- Chemiluminescence, ELISA	[79]
Mefanamic Acid		Lyophilization combined with LC-MS/MS	[65]

**Table 4:** Common PFAS, guideline values, and analytical detection techniques.

Class	Name	EPA maximum contaminant level [80]	Analytical technique	Refs.
Prefluorinated Chemicals	PFOA	4 parts per trillion	1- LC-MS 2- PES followed by LC-MS/MS 3- HPLC-MS/MS 4- UHPLC MS/MS 5- Capillary LC-MS	[9,^64^,81]
	PFOS	4 ppt	1- LC-MS 2- PES followed by LC-MS/MS 3- HPLC-MS/MS 4- UHPLC MS/MS 5- Capillary LC-MS	[9,^64^,82]
	PFNA	1.0 (unitless) Hazard Index	1- HPLC-MS/MS 2- UHPLC MS/MS 3- Capillary LC-MS	[82]
	PFHxS		1- HPLC-MS/MS 2- UHPLC MS/MS 3- Capillary LC-MS	[82]
	PFBS		PES followed by LC-MS/MS	[9]
	HFPO-DA		LC-MS	[83]

## Inorganic compounds

Inorganic water pollutants are chemical compounds that can be present in natural water sources, posing significant risks to human health, wildlife, and ecosystems. Unlike the contamination derived from microorganisms, inorganic pollutants are typically not biodegradable and can persist in the environment for long periods. Heavy metals, such as arsenic, mercury, and cadmium, are examples of inorganic pollutants that can accumulate in the body over time and lead to health complications such as damage to the nervous system, kidneys, and liver. Other inorganic water pollutants are nitrates and nitrites from agricultural runoff and sewage, fluoride from water treatment, and chlorine and chloramines added as disinfectants. Exposure to inorganic pollutants can lead to severe health effects such as cancer, neurological damage, and developmental problems, and impact the quality of water resources, affecting aquatic life and the use of water for drinking and recreational activities. Therefore, identifying and monitoring inorganic water pollutants are crucial for maintaining the safety and sustainability of water resources. Table 5 shows the maximum recommended limits of inorganic water pollutants in drinking and surface water as well as the analytical techniques used for their detection in water.

**Table 5:** Maximum recommended limits of inorganic water pollutants in drinking and surface water and their analytical detection methods.

Name	EPA maximum contaminant level goal (mg/L) [1]	Surface water maximum recommended level (µg/L) [3]	Analytical technique	Ref.
Antimony	0.006	640	Atomic absorption spectrometric	[84]
Arsenic	0	0.14	1- Inductively coupled plasma-mass spectrometry(ICP-MS) 2- Mass spectroscopy 3- X-ray fluorescence spectroscopy 4- Atomic absorption spectroscopy (AAS) 5- Calorimetric 6- Electrochemical detection: amperometric, voltammetry 7- Electrochemical impedance spectroscopy 8- Potentiometric detection	[84,85]
Asbestos (fiber >10 micrometers)	7 million fibers per liter (MFL)			
Barium	2		1- Atomic absorption spectrometric 2- Atomic emission spectrometric	[84]
Beryllium	0.004	64^1^	1- Atomic absorption spectrometric 2- Atomic emission spectrometric (ICP)	[84]
Cadmium	0.005		1- Inductively coupled plasma-mass spectrometry(ICP-MS) 2- Mass spectroscopy 3- X-ray fluorescence spectroscopy 4- Atomic absorption spectroscopy (AAS) 5- Atomic emission spectrometric 6- Electrochemical detection: amperometric, voltammetry, 7- Electrochemical impedance spectroscopy 8- Potentiometric detection	[84], [85], [86]
Chromium (total)	0.1		1- Inductively coupled plasma-mass spectrometry(ICP-MS) 2- Mass spectroscopy 3- X-ray fluorescence spectroscopy 4- Atomic absorption spectroscopy (AAS), 5- Calorimetric 6- Electrochemical detection: amperometric, voltammetry 7- Electrochemical impedance spectroscopy 8- Potentiometric detection	[84,85]
Copper	1.3		1- Inductively coupled plasma-mass spectrometry(ICP-MS) 2- Mass spectroscopy 3- X-ray fluorescence spectroscopy 4- Atomic absorption spectroscopy (AAS), 5- Atomic emission spectrometric 6- Electrochemical detection: amperometric, voltammetry 7- Electrochemical impedance spectroscopy 8- Potentiometric detection 9- CHEMFET	[84], [85], [86]
Cyanide (as free cyanide)	0.2	400	Calorimetric	[84]
Fluoride	4.0		Ion exchange chromatographic	
Lead	0		1- Inductively coupled plasma-mass spectrometry(ICP-MS) 2- Mass spectroscopy 3- X-ray fluorescence spectroscopy 4- Atomic absorption spectroscopy (AAS) 5- Atomic emission spectrometric 6- Electrochemical detection: amperometric, voltammetry 7- Electrochemical impedance spectroscopy 8- Potentiometric detection 9- CHEMFET	[85,86]
Mercury (inorganic)	0.002		1- Inductively coupled plasma-mass spectrometry(ICP-MS) 2- Mass spectroscopy 3- X-ray fluorescence spectroscop 4- Atomic absorption spectroscopy (AAS) 5- Electrochemical detection: amperometric, voltammetry 6- Electrochemical impedance spectroscopy 7- Potentiometric detection	[85]
Nitrate (measured as Nitrogen)	10		Ion exchange chromatographic	[84]
Nitrite (measured as Nitrogen)	1		1- Calorimetric 2- Ion exchange chromatographic	[84]
Selenium	0.05	4200	Atomic absorption spectrometric	[84]
Thallium	0.0005	0.47	Atomic absorption spectrometric	[84]

Although inorganic water pollutants can have long-lasting health effects, they should not be de-prioritized over pollutants that have short-term impacts. Some inorganic pollutants play crucial roles in metabolic activities, while others are purely harmful to the environment. For instance, Hg is present in natural ores and can adsorb onto sediments and rocks due to their high organic carbon content. Even when consumed in trace amounts, it is toxic. Arsenic and selenium are other examples of pollutants that can find their way into water bodies due to disturbances to redox conditions caused by microbial or human activities. As such, these scarcitcan be easily found initially in ground water and later in surface water.

## Particulates

Particulates in water refer to solid or suspended materials that are present in the form of tiny particles. These particles can originate from various sources, including natural processes such as erosion, as well as human activities such as industrial discharges, agricultural runoff, and wastewater effluents. The types of particulates found in water can vary widely and may include sediments, organic matter, microplastics, and pollutants. The detection of particulates in water is of significant importance for several reasons. Firstly, particulates can have adverse effects on water quality, ecosystems, and human health. They can interfere with aquatic life, clog water treatment systems, contribute to the spread of contaminants, and impair the aesthetic appeal of water bodies. Detecting and monitoring particulates helps to assess the overall health and integrity of water resources and supports effective management and remediation strategies.

**Fig. 2:**
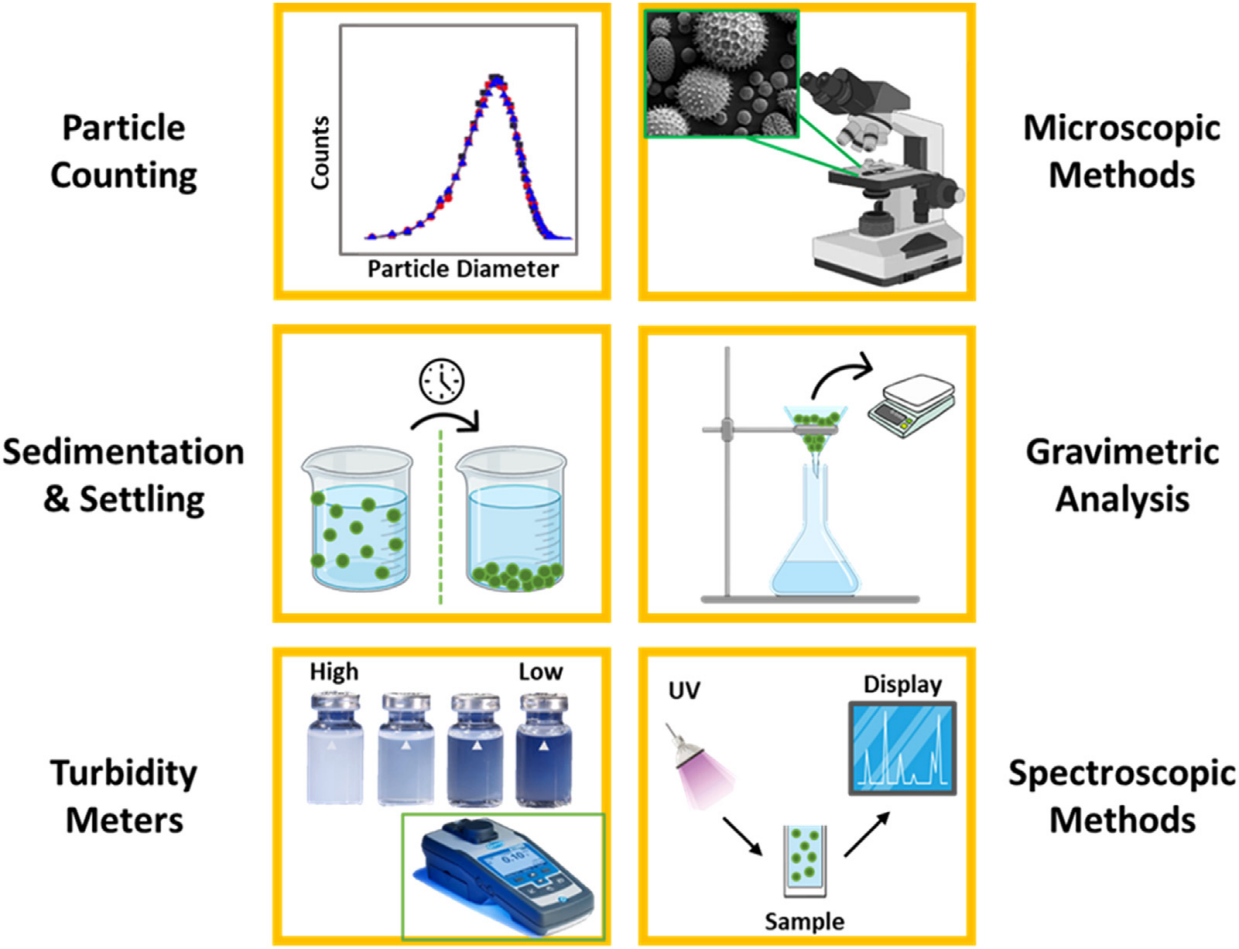
Analytical methods used for the detection of particulates in water.

A range of analytical methods is employed to detect and quantify particulates in water, including turbidity meters, particle counters, filtration and gravimetric analysis, microscopy, spectroscopic methods, and sedimentation and centrifugation (shown in Fig. 2). Turbidity meters are commonly used to measure the cloudiness caused by suspended particles. Particle counters provide information on particle concentration and size distribution. While both turbidity and particle counts are optical measurements of the quantity of particulate material, it is observed that particle counters have become more acceptable analytical tools than turbidity meters [87]. Filtration and gravimetric analysis, on the other hand, is one of the oldest techniques used for quantifying suspended particulate matter. It involves collecting and weighing particulates from filtered water samples. Moreover, microscopy allows for direct visualization and characterization of particles while spectroscopic techniques can identify specific types of particulates based on their optical properties. Finally, sedimentation and centrifugation techniques separate particulates from water for further analysis. These analytical tools play a crucial role in assessing and monitoring the presence and characteristics of particulates in water. By employing these methods, researchers, environmental agencies, and water treatment facilities can gather data necessary for informed decision-making, pollution control, and the preservation of water quality and ecosystem health.

## Microorganisms

Water and wastewater bodies harbor a diverse array of microorganisms, including bacteria, fungi, algae, and protozoa. Understanding the characteristics of microorganisms in such environments is crucial for their detection, surveillance, and treatment. Amongst these microorganisms, bacteria, viruses, and protozoa pose significant risks to human health, with bacteria and viruses being the most extensively investigated. Domestic waste represents a major source of microorganisms in wastewater, although they can also originate from various other sources. These microorganisms can be present in drinking water, seawater, fruits and vegetables, aerosols from irrigation, groundwater, surface water, and marina waters. Industrial activities are another common contributor to microorganism contamination in wastewater. Recognizing the characteristics of microorganisms and the risks they pose to public health is of utmost importance. Infectious diseases, in particular, pose significant risks due to the facilitated spread of pathogens. The incubation period, the time between infection and the onset of symptoms, plays a crucial role in determining the speed of pathogen transmission. For instance, the human immunodeficiency virus (HIV) has a long incubation period, which contributed to its rapid transmission. Wastewater treatment is considered a primary method to address and prevent the transmission of pathogenic diseases. However, it is vital to assess the effectiveness of actual treatment plants, especially in developing countries. This emphasizes the importance of global detection and surveillance studies. In many cases, insufficient information is available about the pathogen or water quality to determine potential risks to the environment and public health. Therefore, the use of reference pathogens, as recommended by the World Health Organization (WHO), becomes necessary [88]. However, this approach can be challenging when dealing with newly emerging enteric pathogens, which often present uncertainties.

Moreover, wastewater treatment plant weaken many viruses and bacteria during the treatment process but sometimes this is not done entirely [89].To combat the presence of pathogens and the unexpected illnesses they may cause, several conventional wastewater disinfection methods have been employed. Chlorination, ozonation, and ultraviolet (UV) irradiation are among the most commonly used disinfection techniques [90]. These methods operate on different principles and may exhibit varying levels of efficiency against specific viruses or pathogenic microorganisms. While chlorine has been the default oxidizer for treatment used for pathogens treatment. This method presents some disadvantages related primarly to environmental issues. This leads to municipalities using ozone as preferred alternative.Dealing with pathogenic diseases that are highly infectious calls for the immediate detection of pathogens in water bodies which need to be localized in order to have a desired effect on the surveillance of pathogens. Thus, continuous detection and monitoring of water bodies is essential to keep track of health-alarming pathogens [3]. Conventional detection methods generally identify pathogens depending on specific constituents. The three general categories of conventional methods are the polymerase chain reaction (PCR), culture and colony counting, and immunology-based methods. Many methods, which lie under these three categories, have been used for the detection of pathogens over the years. Fig. 2 represents a summary of these methods.

### Molecular biology methods

Molecular diagnostic methods primarily rely on the detection of nucleic acids, with the widely used technique being the PCR. It is a well-established method that selectively amplifies specific DNA sequences, making it highly versatile for targeted amplification from any DNA source. Through the use of primers and enzymes, new complementary DNA strands are synthesized, leading to the repetitive amplification of the desired DNA sequence. As a result, the targeted DNA becomes highly concentrated and can be easily detected using labeled probes. These probes generate signals that can be radioactive, colorimetric, fluorometric, or chemiluminescent, depending on the type of molecule under investigation. It is important to note the different variations of PCR techniques available, such as real-time PCR (R-PCR), quantitative real-time PCR (Q-RT PCR), reverse transcriptase PCR (RT-PCR), and multiplex PCR. PCR techniques offer advantages such as speed, specificity, and effectiveness. However, the automated nature of the technique contributes to high running costs, and a sterile environment is essential due to the involvement of biochemical processes. RT-PCR remains a widely utilized and reliable method for virus detection, often serving as a reference in experiments aiming to apply or enhance detection techniques.

### Culture-based methods

**Fig. 3:**
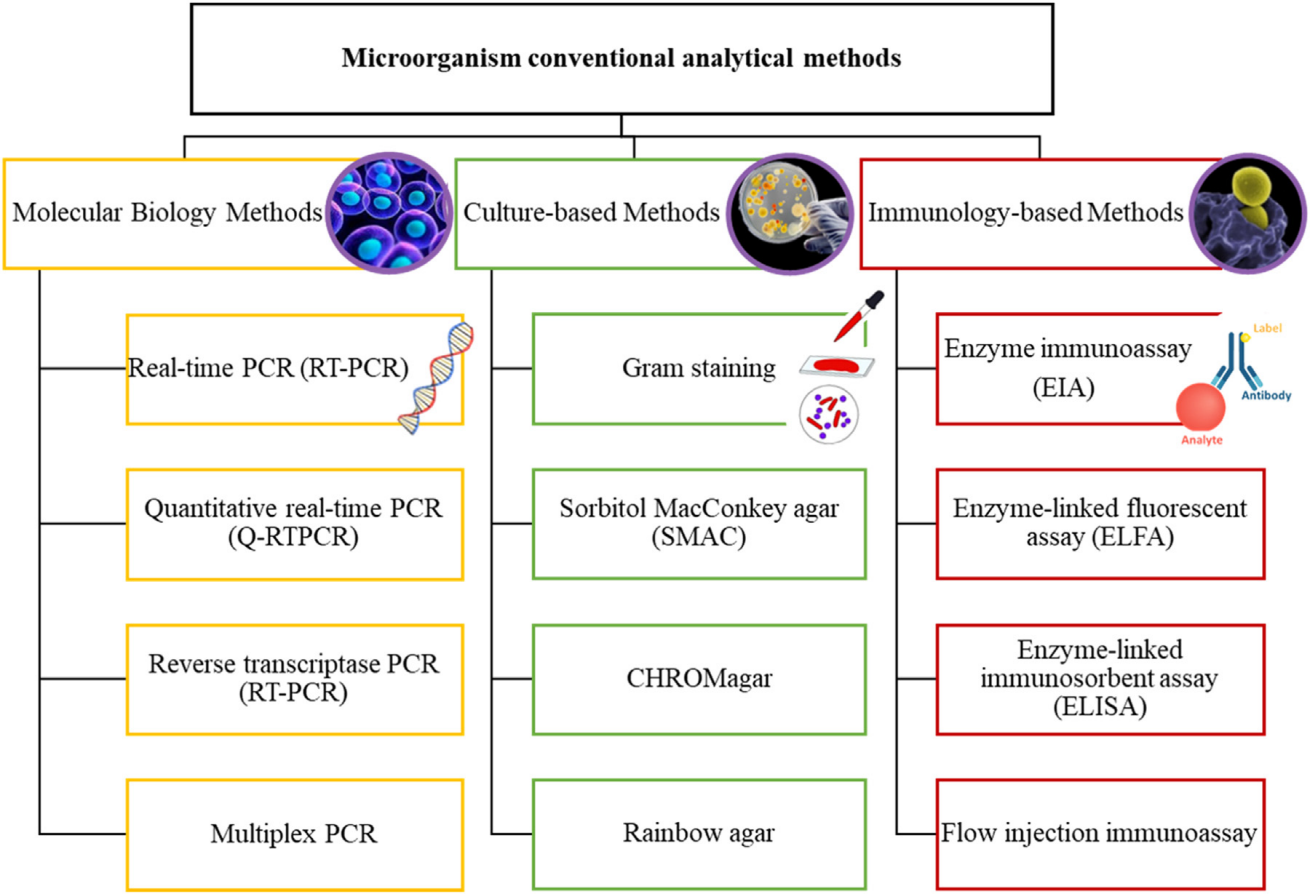
Microorganism conventional analytical methods (developed from Refs. [92,93]).

Among the conventional methods discussed, culture-based methods have the longest history and have been widely employed for microorganism detection. These methods are known for their high success rates, minimal false results, and cost-effectiveness. However, one drawback is the relatively long test duration, typically ranging from 18 to 72 h, due to the slow growth of microorganisms, which can be undesirable in certain situations. Several common culture methods include gram staining, sorbitol MacConkey agar (SMAC), CHROMagar, and rainbow agar. The choice of method often depends on the specific medium used for microorganism growth. For instance, gram staining is used to identify bacteria at suspected infection sites, including the throat, lungs, genitals, or skin wounds. SMAC is commonly used for E. coli detection, relying on sorbitol fermentation, though it tends to yield slower results. On the other hand, CHROMagar employs a chromogenic substance, enabling straightforward color discrimination and finds application in the identification of common yeast species. It is important to note that not all media are suitable for every strain, highlighting the significance of selecting an appropriate medium to obtain desirable results (Fig. 3).

### Immunology-based methods

Immunology-based methods primarily rely on the recognition of pathogens through antigen-antibody bindings. These methods encompass various diagnostic techniques, with enzyme immunoassay (EIA), enzyme-linked fluorescent assay (ELFA), enzyme-linked immunosorbent assay (ELISA), and flow injection immunoassay being among the most commonly used. ELISA, in particular, is a versatile technique employed for detecting specific proteins in biological samples and has undergone numerous enhancements resulting in multiple variations. It is an immune-chemical technique, also known as a solid-phase enzyme immunoassay, as it involves the use of enzyme-linked antigens or antibodies. A positive result is indicated by the formation of a colored product resulting from the reaction between the enzyme-linked protein and the substrate (biological sample). In essence, the ELISA technique can be summarized in three steps: Antigen-antibody reaction, formation of the colored product, and signal detection and quantification. ELISA can be employed in various methodologies such as direct ELISA, indirect ELISA, sandwich ELISA, and competitive ELISA, producing quantitative, qualitative, or semi-quantitative data. The advantages and disadvantages of ELISA vary depending on the specific type being used. For instance, indirect ELISA may exhibit relatively lower specificity compared to other types due to potential cross-reactivity with a secondary antibody [91] (Table 6).

**Table 6:** Comparison between microorganism conventional analytical methods.

Analytical method	Advantages	Disadvantages	Test duration	Cost	Ref.
Molecular biology methods	1- High sensitivity 2- High selectivity	1- Intensive lab work 2- Necessitates a sterile lab environment 3- Costly reagents needed	1–4 h	High	[93]
Culture-based methods	1- High sensitivity 2- High selectivity	1- Intensive lab work 2- Extensive examination 3- Necessitates a sterile lab environment	18–72 h	Low	[87,94]
Immunology-based methods	1- High sensitivity 2- High selectivity	3- Costly analysis 4- Sample enrichment is required	>8 h	High	[88]

## Disinfectants and disinfection by-products

**Fig. 4:**
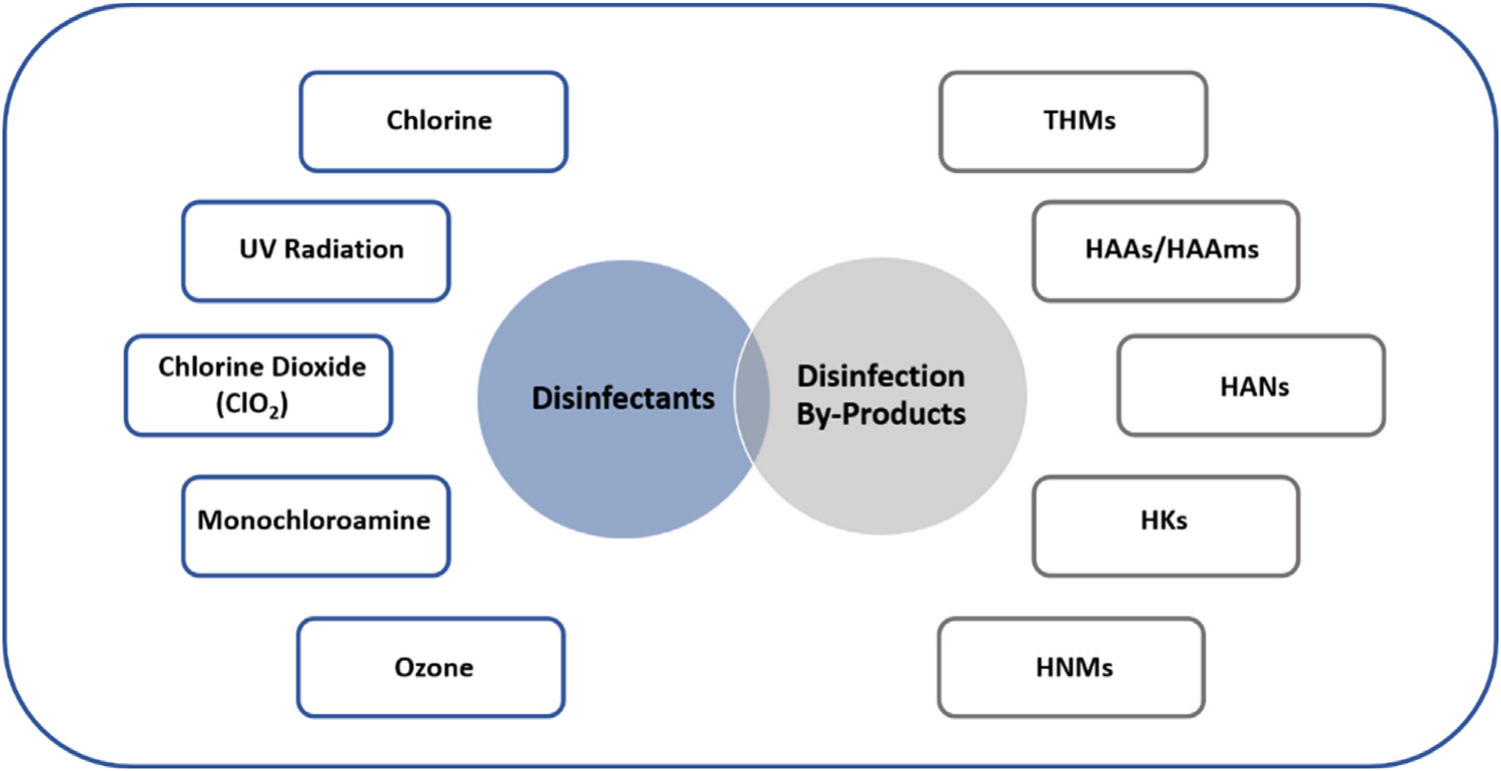
Main types of disinfectants and disinfection by-products found in water.

Disinfectants are commonly used in water treatment and wastewater treatment processes to eliminate harmful microorganisms. The most common disinfectants used worldwide are chlorine, UV radiation, chloride dioxide, monochloroamine, and ozone. Despite the notable success of these disinfectants, their use can inadvertently lead to the formation of DBPs that can contaminate water and wastewater bodies. The most common types of DBPs are THMs, HAAs, and HALs, whereas others include HKs, HNMs, and HAAms. While DBPs generation during disinfection greatly depends on the amount of disinfectant used, researchers have deduced that some DBPs are more toxic than others. For instance, brominated DBPs have been found to be more toxic than chlorinated ones. Nevertheless, multiple research suggests that out of 600 DBPs found in treated water, less than 100 of them are non-toxic in nature. When disinfectants come into contact with organic matter present in water or wastewater, such as natural organic compounds or organic pollutants, chemical reactions occur, resulting in the creation of DBPs. Additionally, exposure to sunlight in surface waters or reservoirs can also contribute to the formation of DBPs through photolysis. Once formed, DBPs can persist in water and wastewater, posing potential health risks to humans and aquatic organisms. Thus, it is crucial to employ careful disinfection practices and monitoring measures to mitigate the presence of DBPs and ensure the safety of water resources. The subsequent sections will discuss the types of DBPs and analytical methods conventionally utilized for their detection in water. Fig. 4 shows the main types of disinfectants and disinfection by-products found in water, while Table 7 shows the multiple methods often employed for the diagnosis of DBPs in water.

**Table 7:** Common disinfectants and disinfection by-products classes, guideline values, and analytical detection techniques.

Class	Name	Drinking water EPA maximum residual disinfectant level goal (mg/L) [1]	Surface water maximum recommended level (µg/L) [3]	Analytical technique	Ref.
Disinfectants	Chloramines (as Cl_2_)	4	–	1- Amperometric titration 2- DPD ferrous titrimetric method 3- DPD colorimetric method 4- UV-Vis Spectrophotometry	[90]
	Chlorine (as Cl_2_)	4	–	1- Amperometric titration 2- DPD ferrous titrimetric method 3- DPD colorimetric method Syringaldazine (FACTS) method 4- Iodometric electrode technique	[91,96]
	Chlorine dioxide (as ClO_2_)	0.8	–	1- Amperometric titration 2- DPD (N,N‑diethyl-p-phenylenediamine) ferrous titrimetric method 3- The Lissamine Green B (LGB WET) colorimetric analytical technique 4- Chronoamperometry	[96,97]
THMs	Bromodichloromethane	0	27	1- GC-MS 2- GC-ECD 3- GC-NiECD 4- Hach THM Plus method 5- P&T/GC-PID-ELCD	[95,^98^,99]
	Bromoform	0	120		
	Dibromochloromethane	0.06	21		
	Chloroform	0.07	2000		
HAAs	Dichloroacetic acid	0	–	1- GC-ECD 2- Hach THM Plus method 3- IC-ESI-MS/MS 4- IC-CD	[95,^98^,99]
	Trichloroacetic acid	0.02	–		
	Monochloroacetic acid	0.07	–		
	Bromoacetic acid	–	–		
	Dibromoacetic acid	–	–		
HANs	–	–	–	GC-ECD	[95,^98^,99]

### Trihalomethanes (THMs)

Trihalomethanes (THMs) are a group of DBPs that are commonly found in chlorinated water. THMs are formed when chlorine, used as a disinfectant in water treatment, reacts with natural organic matter, such as decaying vegetation, and other organic compounds present in water sources. One of the main concerns on THMs is their volatility, which enables their easy vaporization into the air. This property makes inhalation of THMs possible during activities such as showering or using water for household purposes. Additionally, THMs can also be ingested through drinking water or absorbed through the skin during activities like swimming in chlorinated pools.

In terms of chemical structures, THMs are compounds that contain three halogen atoms (typically chlorine, bromine, or a combination of both) bound to a central carbon atom. The most common THMs found in water are chloroform (CHCl3), bromodichloromethane (CHCl2Br), dibromochloromethane (CHClBr2), and bromoform (CHBr3) [95]. Exposure to high levels of THMs over a long period of time has been associated with potential health risks. Studies have suggested links between THM exposure and adverse effects on the liver, kidneys, bladder, and the reproductive system. Some studies have also suggested an increased risk of certain types of cancer, such as bladder cancer and colorectal cancer, although the evidence is not definitive [95]. To address the presence of THMs in drinking water, water treatment facilities employ various strategies, such as optimizing disinfection practices, using alternative disinfectants, or implementing advanced treatment techniques like activated carbon filtration or ozonation, which can help reduce the formation of THMs. Regulatory authorities, such as the Environmental Protection Agency (EPA) in the United States, have set maximum allowable limits for THMs in drinking water to ensure the safety of public water supplies. Regular monitoring and testing of water sources are conducted to ensure compliance with these standards and protect public health.

### Haloacetic acids (HAAs)

Haloacetic acids (HAAs) are another group of DBPs that can be found in chlorinated water. Like many DBPs, HAAs are formed when chlorine or other disinfectants react with organic matter present in water during the disinfection process. HAAs fall under the category of organic DBPs that contain a central carbon atom bonded to a halogen atom and one or more carboxylic acid functional groups. The halogen atoms commonly found in HAAs include chlorine, bromine, or a combination of both. The most prevalent HAAs in drinking water are monochloroacetic acid (CH2ClCOOH), dichloroacetic acid (CHCl2COOH), trichloroacetic acid (CCl3COOH), monobromoacetic acid (CH2BrCOOH), and dibromoacetic acid (CHBr2COOH).

Similar to THMs, exposure to HAAs can occur through ingestion, inhalation, and dermal absorption. However, HAAs are more polar and less volatile than THMs, reducing the risks involved through inhalation. HAAs can be present in drinking water and can also be formed in swimming pools and hot tubs treated with chlorine-based disinfectants. According to Srivastav et al., the factors affecting the generation of HAAs include (1) increased concentration of free chlorine above 3 mg/L, (2) acidic environments of pH 6 or lower, and (3) the presence of hydrophobic natural organic matter [95].

### Haloacetonitrile (HANs)

Research investigations on the presence of Haloacetonitriles (HANs) in water is limited when compared to THMs and HAAs. Mostly, bromochloroacetonitrile, dibromodichloroacetonitrile, and trichloroacetonitrile have been the focal point of investigations.

## Conclusion

Controlling and preventing environmental pollution has risen as a top-tier global priority. These contaminants, existing and emerging ones, consist of organic and inorganic compounds, particulates, microorganisms, and disinfection by-products, all of which pose significant threats to human health, ecosystems, and the environment. Therefore, employing effective monitoring methods is necessary for maintaining water quality whilst implementing corrective actions when needed. In this regard, the development of highly capable analytical technologies is deemed imperative. This paper provides an overview of the lately employed analytical methods for detecting various contaminants in water samples. Chromatography, a powerful analytical technique, plays a pivotal role in environmental monitoring of organic compounds, inorganic compounds, and disinfection by-products by enabling both qualitative and quantitative analysis and precise separation. Gas and liquid chromatography are fundamental chromatographic methods, with LC increasingly replacing GC in many applications due to its advantages. This transition along with continuous research on LC technologies have catalyzed rapid advancements in LC instruments, exemplified by high-performance liquid chromatography, known for its enhanced sensitivity, specificity, and cost-effectiveness. Microorganism control, on the other hand, in water effluents and bodies rely on traditional molecular, culture, and immunology-based methods.

## CRediT authorship contribution statement

**Dana Kadadou:** Methodology, Writing – original draft. **Lina Tizani:** Methodology, Writing – original draft. **Habiba Alsafar:** Writing – review & editing, Validation. **Shadi W. Hasan:** Conceptualization, Writing – review & editing, Validation.

## Declaration of competing interest

The authors declare that they have no known competing financial interests or personal relationships that could have appeared to influence the work reported in this paper.

## Data Availability

All data published in this review is included in this manuscript.
